# Bedaquiline and linezolid regimens for multidrug-resistant tuberculosis: a systematic review and meta-analysis

**DOI:** 10.36416/1806-3756/e20240391

**Published:** 2025-03-18

**Authors:** Mahdis Cheraghi, Mehrnaz Amiri, Sahar Andarzgoo, Fatemeh Zarei, Zahra Sadat Seghatoleslami, Rosella Centis, Dina Visca, Lia D’Ambrosio, Emanuele Pontali, Mohammad Javad Nasiri, Giovanni Battista Migliori

**Affiliations:** 1. School of Medicine, Shahid Beheshti University of Medical Sciences, Tehran, Iran.; 2. Faculty of Nursing and Midwifery, Islamic Azad University, Tehran, Iran.; 3. School of Medicine, Bam University of Medical Sciences, Bam, Iran.; 4. Department of Infectious Diseases, Islamic Azad University, Tehran Medical Branch, Tehran, Iran.; 5. Istituti Clinici Scientifici Maugeri - IRCCS - Tradate, Italia.; 6. Public Health Consulting Group, Lugano, Switzerland.; 7. Servizio di Malattie Infettive, Hospital Galliera, Genova, Italia.

**Keywords:** Linezolid, Tuberculosis, Tuberculosis, multidrug-resistant, Treatment outcome, Systematic review

## Abstract

**Objective:**

Multidrug-resistant tuberculosis (MDR-TB) remains a global public health challenge, complicating treatment strategies and requiring advanced therapeutic approaches. The persistence of MDR-TB has led to a demand for regimens that are more effective in improving treatment outcomes and controlling transmission. This systematic review and meta-analysis sought to examine the efficacy of linezolid (LZD) and bedaquiline (BDQ) in MDR-TB treatment regimens, evaluating their roles in enhancing therapeutic success and informing optimized management of MDR-TB.

**Methods:**

A comprehensive search was conducted across MEDLINE (PubMed), EMBASE, the Cochrane Central Register of Controlled Trials, Scopus, and Web of Science for randomized controlled trials assessing the efficacy of LZD and BDQ in MDR-TB patients up to September 14, 2024. We analyzed treatment outcomes, reporting favorable outcomes (cured and treatment completed) and unfavorable outcomes (death, treatment failure, and loss to follow-up) with a 95% confidence interval.

**Results:**

Our analysis included 11 trials, with a total of 1,999 participants. The findings indicate that BDQ+LZD-containing regimens yield significantly higher favorable treatment outcomes (84.5%; 95% CI, 79.8%-88.2%) and lower unfavorable outcomes (15.4%; 95% CI, 11.6%-20.2%). In contrast, regimens lacking either LZD or BDQ show lower efficacy, with favorable outcomes at 66.8% (95% CI, 59.5%-73.4%) and unfavorable outcomes at 33.0% (95% CI, 25.6%-41.4%).

**Conclusions:**

MDR-TB treatment regimens including BDQ and LZD lead to significantly better patient outcomes. The combined bactericidal and protein synthesis-inhibiting effects of BDQ and LZD create a powerful therapeutic synergy. Adding pretomanid further enhances this effectiveness, highlighting its value in complex cases. Future research should focus on optimizing these regimens for safety and efficacy and explore adjunctive therapies to improve MDR-TB outcomes even further.

## INTRODUCTION

Multidrug-resistant tuberculosis (MDR-TB) represents a significant public health threat. The treatment of MDR-TB needs either prolonged regimens involving multiple antibiotics or shorter regimens that include newer and more expensive (or difficult-to-obtain) drugs. This situation may create considerable challenges for health care systems, especially in low- and middle-income countries, where resources are limited.^1^ ^-^ ^6^ The rising incidence of drug-resistant tuberculosis (DR-TB) not only leads to longer and more costly treatments but also exacerbates health disparities, raising urgent concerns for global health and economic stability.^7^ As health care systems grapple with the dual burden of rising MDR-TB cases and limited resources, the need for effective and accessible treatment options has never been more critical. 

In the last 15 years, the role of linezolid (LZD) and bedaquiline (BDQ) as cornerstones of MDR-TB treatment has emerged, and much has been studied on their safety and efficacy.^8^ ^-^ ^18^ These studies have explored the efficacy of regimens including LZD or BDQ, highlighting their potential to improve treatment outcomes and reduce mortality rates. 

In response to the MDR-TB crisis, the WHO revised treatment guidelines in 2022 to recommend combinations of BDQ, LZD, and pretomanid (Pa), with or without moxifloxacin: the all-oral six-month BPaL and BPaLM regimens.^19^ A further revision occurred in 2024, and the regimens employed in recent clinical trials were recommended as well.^4^ These new treatment protocols always include both drugs (i.e., LZD and BDQ), the goal being to enhance therapeutic outcomes and minimize the economic impact on health care systems. Nevertheless, to our knowledge, no previous systematic review and meta-analysis has investigated the combined role of these two core WHO group A drugs.^20^ ^-^ ^23^ Furthermore, the societal implications of these advancements extend beyond clinical efficacy; they encompass economic factors, access to care, and the broader impact of antimicrobial resistance on public health. 

The objective of this systematic review and meta-analysis was to examine the efficacy of LZD and BDQ in MDR-TB treatment regimens, evaluating their roles in enhancing therapeutic success and informing optimized management of MDR-TB. 

## METHODS

### Definitions

MDR-TB is characterized as a variant of tuberculosis induced by *Mycobacterium tuberculosis* strains that exhibit resistance to at least two fundamental antituberculosis agents: isoniazid and rifampin. The classification of extensively DR-TB (XDR-TB) has undergone significant refinement over time. Initially, XDR-TB was defined as tuberculosis resulting from MDR-TB strains with additional resistance to any fluoroquinolone and at least one of the three second-line injectable agents: kanamycin, amikacin, or capreomycin.^24^ ^,^ ^25^ The 2021 WHO definition of XDR-TB now describes resistance to group A MDR-TB drugs, which include FLQs, LZD, and BDQ.^26^

Prior to 2021, pre-XDR-TB was informally characterized as MDR-TB exhibiting additional resistance to either fluoroquinolones or second-line injectable agents. However, the WHO has revised the definition of XDR-TB to specify that it must include resistance to a fluoroquinolone and either LZD or BDQ, thereby requiring resistance to two of the three group A drugs.^25^ ^-^ ^28^

### Search strategy

We conducted a comprehensive literature search across five major databases-MEDLINE (PubMed), EMBASE, the Cochrane Central Register of Controlled Trials, Scopus, and Web of Science-from January 1, 2009 to September 14, 2024 to identify randomized controlled trials assessing the efficacy of BDQ and LZD and treatment outcomes in DR-TB. The search employed the following search terms in each database separately: “Tuberculosis,” “mycobacterium tuberculosis,” “TB,” “MTB,” “tuberculosis,” “Multi-drug resistant,” “multi drug resistant,” “multi drug-resistant,” “multidrug resistant,” “multi-drug resistance,” “multi drug resistance,” “multi drug-resistance,” “multidrug resistance,” “MDR,” “MDR-TB,” “extensively drug resistant,” “extensively drug-resistant,” “extensively drug resistance,” “extensively drug-resistance,” “extensive drug resistant,” “extensive-drug resistant,” “extensive drug-resistant,” “XDR,” “XDR-TB,” “Pre-XDR,” “Pre-XDR-TB,” “pre-XDR TB,” “Rifampicin Resistant,” “outcome.” 

This study was conducted and reported by the Preferred Reporting Items for Systematic Reviews and Meta-Analyses statement^29^ and was registered with the International Prospective Register of Systematic Reviews (Identifier: CRD42024603453). 

### Study selection

All collected records were consolidated, and duplicates were eliminated with the use of EndNote X8 (Thomson Reuters, Toronto, ON, Canada). Two reviewers independently screened the titles and abstracts, with disagreements being resolved by a third reviewer. They then assessed the full texts of all potentially eligible studies, and any remaining discrepancies were resolved by the third reviewer. 

Eligible studies were selected on the basis of the Population, Intervention, Comparator, and Outcome framework, as follows:

- study design-randomized and nonrandomized controlled trials examining the efficacy of LZD and BDQ in patients with DR-TB
- population-patients ≥ 14 years of age with confirmed DR-TB, including rifampin-resistant tuberculosis, MDR-TB, pre-XDR-TB, and XDR-TB
- intervention-treatment regimens including LZD, BDQ, or both as part of the therapeutic approach to DR-TB
- comparator-comparator arms receiving regimens without LZD and BDQ
- outcome-measured outcomes included treatment success rates, culture conversion, mortality, loss to follow-up, and treatment failure

Articles were excluded if they were cohort studies, case-control studies, cross-sectional studies, case reports/series, reviews, editorials, or conference abstracts. Studies that lacked sufficient data on resistance to LZD and BDQ in DR-TB isolates were also excluded, as were those focusing solely on pregnant women. Additionally, studies not reporting treatment outcomes or using outcomes inconsistent with WHO definitions were omitted. 

### Data extraction

Two authors systematically extracted data into a predefined Microsoft Excel spreadsheet (Microsoft, Redmond, WA, USA). Any discrepancies were resolved with a third reviewer. The extracted data included parameters such as the first author; publication year; study design; study period; country and setting; patient demographics (including age, male count, BMI, prevalence of diabetes mellitus, tobacco use, HIV status, and clinical forms); treatment outcome definitions; number of DR-TB cases; and treatment outcomes. A successful outcome was defined as the sum of “cured” and “treatment completed,” whereas an unsuccessful outcome included “treatment failure,” “loss to follow-up,” and “death.” 

### Quality assessment

The quality of the studies was evaluated by two reviewers using distinct assessment tools, with a third reviewer resolving any inconsistencies. For experimental studies, the Cochrane tool was employed, which assesses various criteria, including random sequence generation, allocation concealment, participant and personnel blinding, outcome assessor blinding, completeness of outcome data, and considerations for selective reporting and other biases. Each study was classified on the basis of the risk of bias: a low risk indicated no concerns; a high risk indicated concerns; and an unclear risk was assigned when information was lacking. 

### Data analysis

Statistical computations were performed with the Comprehensive Meta-Analysis software, version 3.0 (Biostat, Inc., Englewood, NJ, USA). We calculated pooled estimates and 95% confidence intervals for the proportion of patients achieving treatment outcomes. The choice between a random-effects or fixed-effects model was determined by the heterogeneity of effect sizes, as assessed by Cochran’s Q test and the I^2^ statistic. Additionally, publication bias was evaluated by Begg’s test, with a value of p < 0.05 being considered statistically significant. 

## RESULTS

**Figure 1:**
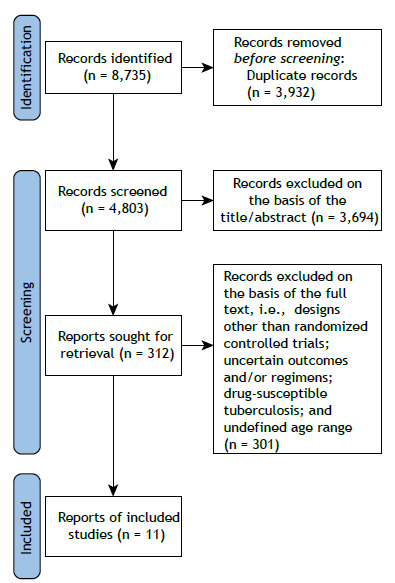
Flow chart of study selection for inclusion in the systematic review and meta-analysis.

As shown in Figure 1, our initial database search identified 8,735 studies. After removing duplicates and conducting title/abstract and full-text screenings, we excluded 8,724 studies, a total of 11 trials including 1,999 patients with various types of DR-TB therefore being included in the final evaluation. All included studies used the previous definition of XDR-TB. The included studies originated from several countries, including India, China, South Korea, and various African nations. The main characteristics of the included studies are shown in Table 1. The mean age of participants was 36.5 years (IQR, 17-71 years). The male-to-female ratio was 1.55, and approximately 19.45% of participants were HIV-positive. Participants were divided into two analytic groups: 839 patients received regimens containing BDQ and LZD, whereas 1,160 patients used regimens that did not include BDQ or LZD. The duration of the studies ranged from 6 months to 24 months, with 1,748 individuals being classified as having rifampin-resistant tuberculosis/MDR-TB and 251 individuals being classified as having pre-XDR-TB/XDR-TB. 

**Table 1:** Characteristics of included experimental studies.

Study ID	Country	DR-TB type	No. of patients	Regimen	Duration	Mean age	Male	HIV+	BMI (kg/m^2^)	DM	Smoking
Nyang’wa et al.^37^ (1)	Uzbekistan, Belarus, and South Africa	MDR-TB	111	BPaL (LZD + Pa + BDQ)	6-9 months	34	57	36	19.9	NM	NM
Nyang’wa et al.^37^ (2)	Uzbekistan, Belarus, and South Africa	MDR-TB	138	BPaLM (LZD + Pa + BDQ + Mfx)	6-9 months	35	77	34	19.7	NM	NM
Nyang’wa et al.^37^ (3)	Uzbekistan, Belarus, and South Africa	MDR-TB	115	BPaLC (LZD + Pa + BDQ + Cfz)	6-9 months	32	76	31	19.4	NM	NM
Yao et al.^38^	China	MDR-TB	34	BDQ+LZD+Lfx+Cs+Cfz/ Lfx+LZD+Cs+Cfz	18 months	43	19	NM	20.3	NM	NM
Nyang’wa et al.^39^	Belarus, South Africa, and Uzbekistan	RR-TB	151	BDQ+LZD+Pa+Mfx	6 months	35	85	38	19.8	NM	NM
Conradie et al.^30^	United Kingdom	XDR-TB/pre-XDR-TB	181	BDQ+LZD+Pa	6 months	36	122	36	20.8	9	113
Goodall et al.^40^ (1)	Ethiopia, Georgia, India, Moldova, Mongolia, South Africa, and Uganda	RR-TB	196	Lfx+Cfz+E+Z/high-dose H + PTO	9 months	> 18	124	27	NM	NM	31
Goodall et al.^40^ (2)	Ethiopia, Georgia, India, Moldova, Mongolia, South Africa, and Uganda	RR-TB	187	Cfz+Z+Lfx/high-dose H + Km	6 months	> 18	115	25	NM	NM	28
Goodall et al.^40^ (3)	Ethiopia, Georgia, India, Moldova, Mongolia, South Africa, and Uganda	RR-TB	127	High-dose Mfx+CFZ+E+Z/+ Km + high-dose H + PTO (intensive phase)	6 months	> 18	77	21	NM	NM	22
Conradie et al.^41^	United Kingdom	MDR-TB/XDR-TB	109	BDQ+LZD+Pa	6-9 months	35	57	56	19.7	NM	NM
Qiujing & Weiwei^42^ (1)	China	MDR-TB	45	Z+Am+Lfx+PTO+E	21 months	45.3	30	0	NM	NM	NM
Qiujing & Weiwei^42^ (2)	China	MDR-TB	45	Am+E+Z+Mfx+PTO+E	21 months	44.5	28	0	NM	NM	NM
Du et al.^43^ (1)	China	MDR-TB	67	Cm+CFZ+Cs+Lfx+PTO+Z	12 months	37.9	44	0	19.8	1	NM
Du et al.^43^ (2)	China	MDR-TB	68	Cm+E+Cs+Lfx+PTO+Z+E	12 months	39	45	0	20.1	3	NM
Duan et al.^44^ (1)	China	MDR-TB	66	Am/Cm+Lfx+Z+E+PAS+/PTO+Amx/Clv+CFZ	24 months	36.8	44	0	19.9	2	NM
Duan et al.^44^ (2)	China	MDR-TB	74	Am/Cm+Lfx+Z+E+PAS+/PTO+Amx/Clv+Lfx+Z+E+PAS/PTO+Amx/Clv	24 months	36.4	44	0	19.8	2	NM
Nunn et al.^45^	United Kingdom	RR-TB	253	short regimen: Mfx+CFZ+E+Z+Km+H+PTO	20 months	> 18	151	85	NM	NM	NM
Tang et al.^46^	China	XDR-TB	32	PTO+Z+Mfx/Gfx/Lfx/ PAS+CPM+Am+CFZ+CLA	24 months	43	21	0	19.6	6	NM

DR-TB: drug-resistant tuberculosis; DM: diabetes mellitus; MDR-TB: multidrug-resistant tuberculosis; XDR-TB: extensively drug-resistant tuberculosis; RR-TB: rifampin-resistant tuberculosis; Am: amikacin; PTO: prothionamide; Cm: capreomycin; Lfx: levofloxacin; Z: pyrazinamide; PAS: para-aminosalicylic acid; Pa: pretomanid; Amx/Clv: amoxicillin/clavulanate; Cfz: clofazimine, LZD: linezolid; BDQ: bedaquiline; Mfx: moxifloxacin; E: ethambutol; H: isoniazid; Km: kanamycin; Gfx: gatifloxacin; CPM: chlorpheniramine; CLA: clarithromycin; Cs: cycloserine; and NM: not mentioned.

In regimens that included both BDQ and LZD, Pa was the most frequently used drug, often accompanied by moxifloxacin and clofazimine. In contrast, non-BDQ/LZD regimens commonly featured levofloxacin, clofazimine, and ethambutol, along with additional combinations that included amikacin and capreomycin. These non-BDQ/LZD regimens generally exhibited varying levels of efficacy, typically resulting in higher rates of unfavorable outcomes in comparison with BDQ-LZD combinations. 

### Quality of the included studies

The quality of the included clinical trials was assessed with the Cochrane tool. The checklist showed that the included studies had a low risk of bias (Table 2). Of the included studies, the one conducted by Conradie et al.^30^ showed a high risk of blinding of participants, personnel, and outcome assessment. 

**Table 2:** Quality assessment of included experimental studies (the Cochrane tool).

Author	Random sequence generation	Allocation concealment	Blinding of participants and personnel	Blinding of outcome assessment	Incomplete outcome data	Selective reporting	Other bias
Nyang’wa et al.^37^	Low risk	High risk	High risk	High risk	Low risk	Low risk	Low risk
Yao^38^	Low risk	High risk	High risk	High risk	Low risk	Low risk	Low risk
Nyang’wa^39^	Low risk	High risk	High risk	High risk	Low risk	Low risk	Low risk
Conradie et al.^30^	Low risk	Low risk	Low risk	Low risk	Low risk	Low risk	Low risk
Goodall et al.^40^	Low risk	High risk	High risk	High risk	Low risk	Low risk	Low risk
Conradie et al.^41^	High risk	High risk	High risk	High risk	Low risk	Low risk	Low risk
Qiujing & Weiwei^42^	High risk	High risk	High risk	High risk	Low risk	Low risk	Low risk
Du et al.^43^	Low risk	High risk	High risk	High risk	Low risk	Low risk	Low risk
Duan et al.^44^	Low risk	High risk	High risk	High risk	Low risk	Low risk	Low risk
Nunn et al.^45^	Low risk	High risk	High risk	High risk	Low risk	Low risk	Low risk
Tang et al.^46^	Low risk	High risk	High risk	High risk	Low risk	Low risk	Low risk

### Pooled treatment outcomes in the BDQ-LZD group

Five studies featured regimens that included both BDQ and LZD. In the cohort of 839 patients receiving the BDQ-LZD regimen, 645 achieved favorable outcomes, whereas 114 experienced unfavorable outcomes. Favorable outcomes were classified as either cured or treatment completed, whereas unfavorable outcomes included treatment failure, death, and loss to follow-up. 

**Figure 2:**
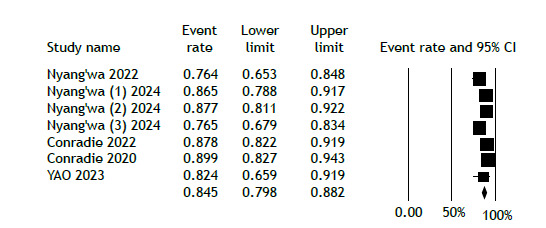
Favorable treatment outcome in the linezolid-bedaquiline group.

**Figure 3:**
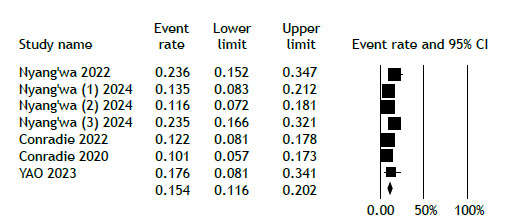
Unfavorable treatment outcome in the linezolid-bedaquiline group.

Figure 2 displays the analysis of favorable outcomes among participants receiving the BDQ-LZD regimen, demonstrating an overall favorable outcome rate of 84.5% (95% CI, 79.8%-88.2%). In contrast, the overall unfavorable outcomes for patients on this regimen were reported at 15.4% (95% CI, 11.6%-20.2%; Figure 3). Because of the limited duration of the trials, no temporal trend was observed. 

### Pooled treatment outcomes in the non-BDQ/LZD group

**Figure 4:**
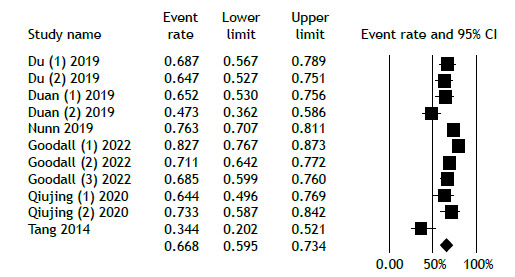
Favorable treatment outcome in the non-bedaquiline/linezolid group.

**Figure 5:**
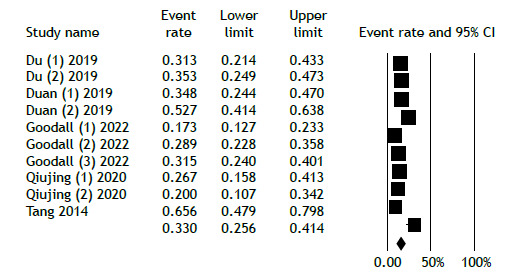
Unfavorable treatment outcome in the non-bedaquiline/linezolid group.

Six studies involved regimens that did not include BDQ or LZD. Of the 1,160 patients in this group, 816 achieved favorable outcomes. Figure 4 illustrates the analysis of favorable outcomes for individuals receiving regimens without BDQ and LZD, revealing an overall favorable outcome rate of 66.8% (95% CI, 59.5%-73.4%). Conversely, the overall unfavorable outcomes among patients on these regimens were reported at 33.0% (95% CI, 25.6%-41.4%; Figure 5). Because of the limited duration of the trials, no temporal trend was observed. 

## DISCUSSION

The results of this analysis clearly demonstrate that treatment regimens incorporating BDQ and LZD yield significantly better patient outcomes than do those that do not include both agents. The BDQ+LZD-based regimens achieved an impressive favorable outcome rate of 84.5%, whereas the non-BDQ/LZD group had a substantially lower favorable outcome rate of 66.8%. This pronounced difference highlights the superior bactericidal and sterilizing activity of BDQ and LZD, both of which are critical in effectively treating MDR-TB. 

One of the key advantages of BDQ and LZD lies in their ability to target different sites of the bacterial cell, resulting in rapid bacterial clearance, reduced risk of resistance, and reduced risk of relapse. BDQ disrupts ATP synthase, essential for *M*. *tuberculosis* survival, whereas LZD inhibits protein synthesis, together creating a potent synergistic effect that accelerates treatment response. 

The association of BDQ with LZD is not however sufficient for the effective treatment of MDR-TB strains. They need accompanying drugs, such as fluoroquinolones, Pa, and clofazimine. Although it was beyond the scope of this review to investigate the best companions for BDQ and LZD, some considerations can be made. 

Although injectables (aminoglycosides) now seem obsolete, fluoroquinolones and Pa appear to play an important role in building a BDQ+LZD-based regimen for MDR-TB treatment. Although fluoroquinolones contribute to the bactericidal activity of non-BDQ/LZD regimens, they show reduced efficacy in comparison with combinations of BDQ and LZD. When Pa is added to BDQ-LZD regimens, it further enhances treatment efficacy by disrupting *M*. *tuberculosis* cell wall synthesis and targeting persister cells, which are challenging to eradicate even with standard regimens for drug-susceptible tuberculosis. 

Although BDQ, LZD, and Pa are crucial for MDR-TB treatment, their toxicity profiles warrant careful monitoring. BDQ has been associated with QT interval prolongation, which poses a risk of cardiac arrhythmias, particularly in patients with preexisting heart conditions or those on concurrent QT-prolonging drugs.^11^ ^,^ ^30^ ^-^ ^32^ Although LZD is effective, it is linked to bone marrow suppression, peripheral neuropathy, and optic neuropathy, especially when used for long periods of time. Recent studies have emphasized that monitoring blood counts and neurological symptoms can help mitigate these risks.^33^ ^-^ ^35^ Although Pa has been less studied, it has been associated with hepatotoxicity and gastrointestinal side effects, which are exacerbated in patients with liver conditions.^36^ These findings underscore the importance of balancing efficacy with safety through vigilant toxicity monitoring to optimize patient outcomes while minimizing adverse effects. 

Our study has some notable limitations. First, the variability in study designs, sample sizes, and treatment protocols across the included trials may introduce heterogeneity, affecting the generalization of our findings. Second, data on patient demographics and comorbidities were sometimes insufficient, limiting our ability to fully evaluate their influence on treatment outcomes. Although we focused on the efficacy/effectiveness of BDQ and LZD, this analysis may overlook the potential synergistic effects of other essential drugs in combination therapies, which could significantly impact patient success. In particular, no study included delamanid in any treatment regimen. Additionally, because of the dynamic nature of MDR-TB treatment guidelines, ongoing research is needed to assess the long-term effectiveness of these regimens in real-world settings. 

Interestingly, additional information will be available when the full results of two major clinical trials have been published. These two studies proposed a series of different and unusual but effective BDQ-LZD combinations of drugs without Pa, but with fluoroquinolones and/or delamanid.^6^ The results, which are probably stunning, have been disclosed to the WHO, leading to a new recommendation for the treatment of patients with MDR-TB.^4^

In conclusion, the present study demonstrates that treatment regimens incorporating BDQ and LZD offer significantly improved outcomes for MDR-TB patients in comparison with regimens without these agents. The synergy between the bactericidal effects of BDQ and the protein synthesis inhibition by LZD provides a powerful approach to combatting *M*. *tuberculosis*, resulting in higher rates of favorable outcomes. The addition of Pa further enhances the effectiveness of BDQ-LZD regimens, reinforcing its value in complex MDR-TB cases. Future research should aim to refine these regimens, balancing safety and efficacy, and explore adjunctive therapies to further improve MDR-TB treatment outcomes. 
